# Deciphering Soil Keystone Microbial Taxa: Structural Diversity and Co-Occurrence Patterns from Peri-Urban to Urban Landscapes

**DOI:** 10.3390/microorganisms13081726

**Published:** 2025-07-24

**Authors:** Naz Iram, Yulian Ren, Run Zhao, Shui Zhao, Chunbo Dong, Yanfeng Han, Yanwei Zhang

**Affiliations:** 1 Institute of Fungus Resources, Department of Ecology, College of Life Sciences, Guizhou University, Guiyang 550025, China; iramnaz1996@126.com (N.I.); renyulian0411@163.com (Y.R.); zhaozhaorr@163.com (R.Z.); zs15185344163@126.com (S.Z.); cbdong@gzu.edu.cn (C.D.); 2 Guizhou Key Laboratory of Agricultural Microbiology, Guiyang 550025, China; 3 Key Laboratory of Development and Utilization of Biological Resources in Colleges and Universities of Guizhou Province, Guizhou Education University, Guiyang 550018, China; 4 Key Laboratory of Ecology and Management on Forest Fire in Higher Education Institutions of Guizhou Province, Guizhou Education University, Guiyang 550018, China

**Keywords:** keystone taxa, community assembly, community stability, co-occurrence patterns, urbanization

## Abstract

Assessing microbial community stability and soil quality requires understanding the role of keystone microbial taxa in maintaining diversity and functionality. This study collected soil samples from four major habitats in the urban and peri-urban areas of 20 highly urbanized provinces in China using both the five-point method and the S-shape method and explored their microbiota through high-throughput sequencing techniques. The data was used to investigate changes in the structural diversity and co-occurrence patterns of keystone microbial communities from peri-urban (agricultural land) to urban environments (hospitals, wastewater treatment plants, and zoos) across different regions. Using network analysis, we examined the structure and symbiosis of soil keystone taxa and their association with environmental factors during urbanization. Results revealed that some urban soils exhibited higher microbial diversity, network complexity, and community stability compared to peri-urban soil. Significant differences were observed in the composition, structure, and potential function of keystone microbial taxa between these environments. Correlation analysis showed a significant negative relationship between keystone taxa and mean annual precipitation (*p* < 0.05), and a strong positive correlation with soil nutrients, microbial diversity, and community stability (*p* < 0.05). These findings suggest that diverse keystone taxa are vital for sustaining microbial community stability and that urbanization-induced environmental changes modulate their composition. Shifts in keystone taxa composition reflect alterations in soil health and ecosystem functioning, emphasizing their role as indicators of soil quality during urban development. This study highlights the ecological importance of keystone taxa in shaping microbial resilience under urbanization pressure.

## 1. Introduction

Urbanization significantly changes the earth’s surface landscape, posing a major threat to global biodiversity [1,2]. It causes dramatic changes to the terrestrial landscapes, including surface vegetation, hydrology, and soil, with consequent impacts on biodiversity [3,4]. Managing urbanization’s threats to biodiversity is a key concern for ecologists. Urbanization has a major effect on soil microbial communities, which changes important ecosystem services and processes like carbon storage and nutrient cycling. This has consequences for global biogeochemical cycles and climate regulation [5]. Human activities in urban settings, such as sewage treatment [6], greening places [7,8], park development [9], and agricultural land management [10], influence microbial communities. This may potentially reduce soil microbial biomass [11,12] and lead to the disappearance of indigenous microorganisms [13]. Urbanization has a substantial effect on soil microbiota in many parts of the world, changing the variety, composition, and function of microorganisms. The largest Arctic city in Russia, Murmansk, has seen significant urbanization, which has changed the soil’s characteristics and microbiota. Murmansk’s urban soils show increases in carbon and nutrient content as well as pH shifts towards neutral, both of which typically encourage microbial growth [14]. It has been demonstrated that the soil microbiome in Latin America is shaped by the urbanization gradient [15]. Urban biodiversity and soil microbial communities are directly impacted by urbanization processes such as habitat loss and the eradication of native plant species [12]. Despite this, only a small fraction of soil studies had focused on urban greenfield soil [4]. For example, in the city of Beijing, China, soil bacterial diversity decreased then increased from the city center to the suburbs [16], and parks with longer establishment histories had higher soil microbial diversity [17]. Understanding the effects of urbanization on soil microbes is vital for eco-city planning and biodiversity conservation [4].

In microbial communities, keystone taxa are critical for community stability and can trigger dramatic changes in community structure and function if removed [18,19]. At present, the utilization of co-occurrence networks to identify keystone taxa has been accepted by a broad scientific consensus, and keystone taxa can be identified with high accuracy using high average degree, high centrality, and low betweenness centrality [20,21]. The keystone taxa predominate in shaping the co-occurrence network across entire communities, and removal of these keystone taxa results in the collapse of the co-occurrence network [22,23]. However, current understanding of the diversity, community assembly, and co-occurrence patterns of keystone microbial communities in the background of urbanization remains limited. Therefore, it is essential to indicate the impact of urbanization on the keystone taxa of soil microorganisms through microbial co-occurrence networks; this can provide valuable insights into the contribution of soil microbial communities to the balance of urban ecosystems.

This study focused on 20 highly urbanized cities and adopted the spatial gradient method instead of the time sequence approach to analyze areas such as hospitals, sewage treatment plants, and zoos, as well as peri-urban areas like agricultural fields. High-throughput sequencing techniques combined with network analysis were employed to explore keystone microbial interactions and community stability. Understanding microbial communities and finding keystone taxa are two advantages of network analysis. Networks make it possible to identify keystone taxa, which are highly connected “hubs” within the network that reflect their important role in the microbial community, regardless of their abundance [24]. These taxa can affect the microbiome by producing metabolites that change the composition of microorganisms or by influencing the abundance of other species [24]. Keystone species are disproportionately crucial to the stability and functioning of ecosystems [23]. Keystone species are more observable in co-occurrence networks than in interaction networks because they typically have a high mean degree, low betweenness centrality, high closeness centrality, and high transitivity [20].

Furthermore, partial least squares path modeling (PLS-PM) was employed to assess the contribution of key biotic and abiotic factors to community stability. The study aimed to determine changes in the community structure of keystone taxa and their impact on the complexity and stability of microbial co-occurrence networks, as well as the relationship between the environmental factors and the stability of keystone microbial communities. This formed the central hypothesis of the study. The findings provide a theoretical foundation for the evaluation and prediction of soil environmental changes, which are crucial for the protection of soil resources and the management of soil quality during urbanization.

## 2. Materials and Methods

### 2.1. Study Sites and Sample Collection

Soil samples were collected in July 2021 from 20 highly urbanized provinces typical of north China (Beijing, Tianjin, Shanxi, Hebei), east China (Shandong, Jiangsu, Zhejiang), and central China (Henan) (Supplementary Table S1 and Supplementary Figure S1). Each sample collected was approximately 100 g. A total of four major habitats in urban (hospitals, sewage treatment plants, and zoos)–peri-urban areas (agricultural land) were selected as sampling sites. The urban and peri-urban greenspace soils were chosen because they are likely to be rich in microscopic organisms and affected by artificial interference. Samples were collected from three randomly selected sampling points in each plot using a mixture sampling method. For each sampling point, a plot of approximately 100 m^2^ was selected and 15 soil samples were randomly collected based on the size and shape of the plot, using both the five-point method and the S-shape method (peri-urban agricultural land). Surface vegetation and approximately 1 cm of cover material (i.e., gravel) were removed prior to sampling. Soil was then collected from a depth of 0 to 10 cm. Gravel and debris, such as plant residues, were removed before mixing the soil samples from 15 collection points, resulting in a total of 240 mixed soil samples. At the urban sites, zoo samples were taken from areas where various animals were located at the sampling sites. Areas of the hospital with higher population density were selected for greenspace soil sampling, whereas greenspace soil samples were collected near the wastewater treatment plants. For the peri-urban urban sites, common agricultural areas such as corn fields were selected.

The soil samples were divided into two parts. One part was stored at −80 °C for DNA extraction, and the other part was used to determine soil abiotic factors. The biological replicates of each sample were completed four times.

### 2.2. Determination of Abiotic Factors

The study determined total soil nitrogen (TN) using the sulfuric acid–perchloric acid digestion Kjeldahl method [25]. Total phosphorus (TP) was measured using the H_2_SO_4_-HCLO_4_-molybdenum-antimony resistance colorimetric method [26], while total potassium (TK) was assessed using the NaOH fusion flame spectrophotometry [26]. Available nitrogen (AN) was determined using the alkaline hydrolysis diffusion method [27], and available phosphorus (AP) was measured by UV spectrophotometry with 0.5 mol/L NaHCO_3_ extraction [28]. Available potassium (AK) was extracted with 1 mol/L neutral NH_4_OAC and measured using flame photometry [26]. Soil organic carbon (SOC) was analyzed using the dichromate oxidation with external heating procedure [27]. Finally, soil pH was determined with a glass electrode in a 1:2.5 soil-to-water solution (g/mL). In addition, mean annual precipitation (MAP) and mean annual temperature (MAT) data were obtained from the China Meteorological Data Network (http://data.cma.cn, accessed on 1 December 2021) and the Spatial Data Consortium database (CGIAR-CSI; http://www.cgiar-csi.org/, accessed on 10 December 2021) [23]. All soil physicochemical parameters and climatic data are presented in a Supplementary Table S2.

### 2.3. DNA Extraction and Illumina Novaseq Sequencing

Soil keystone bacterial and fungal communities were analyzed using high-throughput amplicon sequencing. DNA extraction and Illumina Novaseq sequencing procedures were described in a previous study [23]. Briefly, total DNA was extracted from 0.5 g of fresh soil using the FastDNA SPIN kit (MP Biochemicals, Solon, OH, USA) following the manufacturer’s instructions. The bacterial V3-V4 region was amplified using primers 338F and 806R, while the fungal community was amplified using ITS1 and ITS2 primers [29]. Sequencing was performed on the Illumina Miseq PE300 platform (Beijing Biomarker Technologies Co., Ltd., Beijing, China). The comparison threshold was set at 70%. A total of 1,600,502 and 1,599,223 pairs of reads for bacteria and fungi were sequenced, respectively. After quality control and chimera removal, sequences with at least 97% similarity were clustered into operational taxonomic units (OTUs) using UPARSE software 7.0.1001 [30]. Representative OTUs were obtained after chimera filtering and then annotated using the Silva 16S rRNA database (v138) and RDP classifier [31]. Subsequent analyses were based on the resulting OTUs table. A total of 1,594,774 clean reads with an average of 79,739 clean reads were generated for bacteria, while 1,593,537 clean reads with an average of 79,677 clean reads were generated for fungi. All raw sequencing data has been deposited in the NCBI SRA database under access number PRJNA841243.

### 2.4. Definition of Keystone Bacterial and Fungal Taxa

In this study, network analysis was used to identify and construct keystone bacterial and fungal taxa. First, Spearman’s correlation coefficients (r ≥ |0.6|, *p* < 0.05) were calculated between OTUs within bacterial or fungal communities using the “psych” package in version R 4.3.2. OTUs with relative abundance below 0.01% were filtered out prior to correlation analysis. The *p*-values were then adjusted using the false discovery rate (FDR) method. Subsequently, separate co-occurrence networks for bacterial and fungal communities were constructed using the igraph package. Following network construction, we calculated the topological properties of bacterial and fungal communities, including total nodes, total edges, average degree, clustering coefficient, and modularity, to assess the internal structure of the networks among different taxa in the greenspace areas of the zoos, hospitals, wastewater treatment plants, and farmland. Furthermore, within-module connectivity (Zi) and among-module connectivity (Pi) values were calculated using the “WGCNA”, “igraph”, and “ggplot2” packages to partition the modules. Module hubs, connectors, network hubs, and peripheral nodes were identified based on the Zi-Pi scatter plot. OTUs with Zi > 2.5 and Pi < 0.62 are identified module hubs (nodes with high connectivity within the module). OTUs with Zi < 2.5 and Pi > 0.62 are identified as connected nodes in the network (nodes with high connectivity between two modules). OTUs with Zi < 2.5 and Pi < 0.62 were identified as peripheral nodes (nodes with low connectivity within and between modules) [32]. Apart from peripheral nodes, module hubs, connectors, and network hubs were identified as potential keystone taxa due to their critical roles in the network topology [33].

### 2.5. Community Stability Index

The average variation degree (AVD) was used to evaluate microbial community stability [32,34]. A lower AVD value indicates higher microbial stability. To calculate the variability of each OTU, we used the following Equation (1):

(1) $$\left|a_{i}\right|=\frac{\left|x_{i}-\bar{x_{i}}\right|}{\delta_{i}}$$

In Equation (1), the variation degree for an OTU is denoted as *ai*, where *xi* is the rarefied abundance of the OTU in a given sample; *xi* is the average rarefied abundance of the OTU within a sample group; and *δi* is the standard deviation of the rarefied abundances of the OTU within that group.

The AVD values were calculated using Equation (2), where *k* is the number of samples in a sample group, and *n* is the number of OTUs in each sample group [32,34].

(2) $$AVD=\frac{\sum_{i=1}^{n}\frac{\left|x_{i}-\bar{x_{i}}\right|}{\delta_{i}}}{k\times n}$$

### 2.6. Partial Least Squares Path Model (PLS-PM) Analysis

Partial least squares path modeling (PLS-PM) was used to analyze the effects of soil nutrients (total phosphorus, total nitrogen, total potassium, organic carbon, available nitrogen, available phosphorus, and available potassium), pH, climatic factors (mean annual precipitation and mean annual temperature), and biological factors (community diversity and keystone taxa) on the stability of microbial communities. The “plspm” package in version R 4.3.2 was used to construct the model, comprising latent and observed variables. Path coefficients (i.e., standardized partial regression coefficients) and the significance of each path were calculated to assess the strength and direction of the relationship between the latent and observed variables, including both direct and indirect effects. Finally, the *R*^2^ value indicates the proportion of variance explained by independent latent variables, while the Goodness of Fit index was used to evaluate the overall performance of the model [35].

### 2.7. Statistical Analysis

One-way ANOVA (SPSS 22.0) was used to test for significant differences in alpha diversity and the relative abundance of keystone taxa across the four habitats. FAPROTAX was used to predict the functional profiles of bacterial taxa, while FUNGuild (http://stbates.org/funguild_db.php) was used to predict the functional profile of fungal taxa [36]. Levin’sniche-width index was applied to calculate the niche breadth of each keystone microbial community [23,37].

## 3. Results

### 3.1. Soil Physicochemical and Climatic Characteristics

Our findings revealed that peri-urban (agricultural) soil contained higher levels of nutrients (C, N, P, and K) compared to urban soils (hospitals, sewage treatment plants, and zoos). Soil pH ranged from 3.27 to 8.53, with over 90% of the samples exhibiting weakly acidic conditions. Additionally, cities in eastern China (Jiangsu and Zhejiang) reported higher mean annual temperatures and precipitation than other cities. Significant variations in soil nutrient content, pH, mean annual temperature, and precipitation were observed across different cities (Supplementary Table S2).

### 3.2. Identification and Co-Occurrence Networks of Potential Keystone Taxa

**Figure 1:**
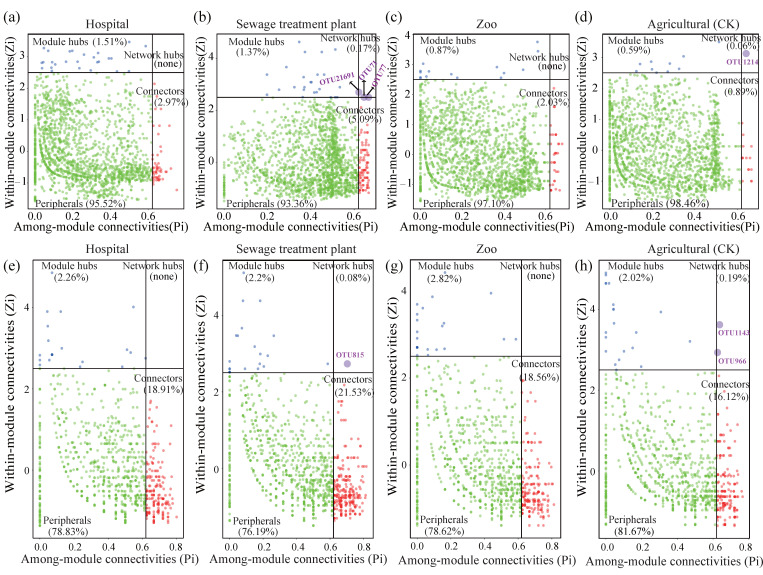
Identification of keystone taxa in urban and peri-urban green soil microbial communities based on their network topological features. Module hubs are identified as Zi > 2.5 and Pi < 0.62, connectors are identified as Zi < 2.5 and Pi > 0.62, and network hubs are identified as Zi > 2.5 and Pi > 0.62. The transverse coordinate is the inter-module connectivity. The vertical coordinate is the degree of intra-module connectivity. Different-colored dots represent nodes with different attributes. (**a**–**d**) Module hubs and network hubs of bacterial communities in Hospital, Sewage treatment plant, Zoo and Agricultural soils, respectively; (**e**–**h**) Module hubs and network hubs of fungal communities in Hospital, Sewage treatment plant, Zoo and Agricultural soils, respectively.

Generally, nodes with within-module connectivity (Zi) > 2.5 or among-module connectivity (Pi) > 0.62 are considered as keystone species. In this study, peripheral nodes accounted for over 90% of bacterial communities in both urban and peri-urban soil (Figure 1a–d), while they represented more than 76% of fungal communities (Figure 1e–h). Bacterial and fungal network hub taxa were detected only in urban (sewage treatment plants) and peri-urban (agricultural) areas and were absent in other greenspace areas (Figure 1). Specifically, the bacterial network hubs in the urban areas (sewage treatment plants) were OTU21691, OTU71, and OTU77, while in the peri-urban areas (farmland), OTU1214 was identified. For fungi, the network hub in the urban areas was OTU815, while in the peri-urban areas they were OTU1143 and OTU966. These OTUs appear to play important roles in the co-occurrence networks. Moreover, the number of connectors and module hubs was substantially higher in the hospitals (51, 26), wastewater treatment plants (89, 24), and zoos (35, 15) compared to the agricultural locations (15, 10) (Figure 1a–d). Similarly, the number of fungal connectors and module hubs was significantly greater in the hospitals (184, 22), zoos (254, 26), and wastewater treatment plants (191, 29) than in the agricultural sites (168, 21) (Figure 1e–h). In conclusion, although the agricultural sites had the highest proportion of bacterial and fungal peripheral nodes, they showed the lowest numbers of module hubs and connectors (Figure 1).

**Figure 2:**
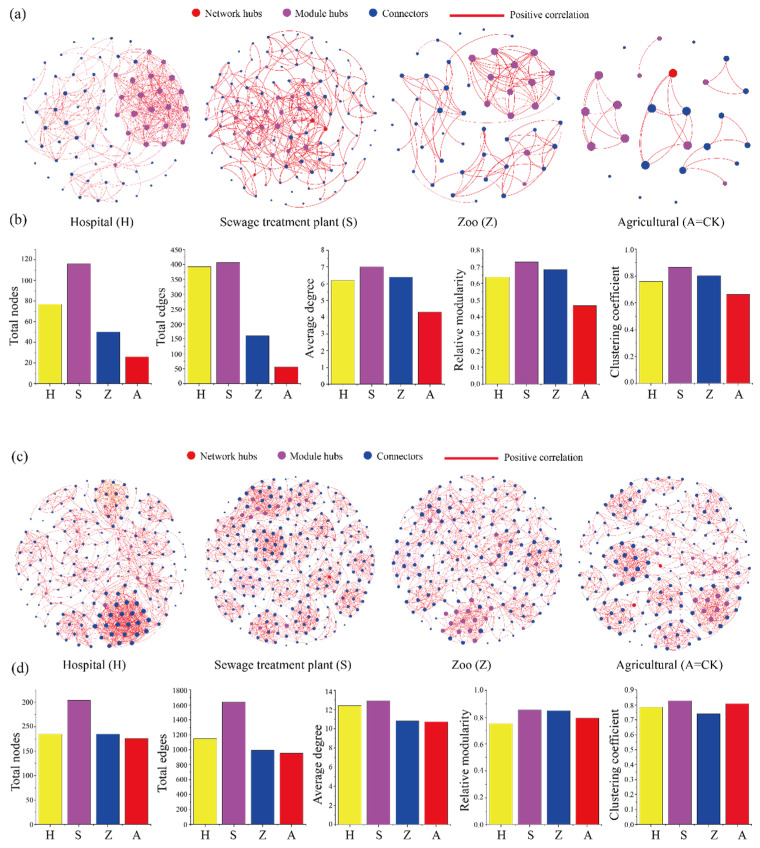
Co-occurrence network patterns and topological properties of keystone taxa in urban (hospitals, sewage treatment plants, and zoos) and peri-urban (agricultural) soils. The node size varies according to the degree value of the node, with red circles representing network hubs, purple circles representing module hubs, and blue circles representing connectors. The red line represents the positive correlation between the keystone taxa. (**a**,**c**): Co-occurrence networks of keystone bacterial and fungal communities in urban and peri-urban soils. (**b**,**d**): Topological properties of the keystone bacterial and fungal community co-occurrence networks in urban and peri-urban soils. H: hospital; S: sewage treatment plant; Z: zoo; A: agricultural.

Co-occurrence network patterns of keystone taxa differ between urban and peri-urban areas (Figure 2). The hospital, sewage treatment plant, and zoo soils showed higher values for nodes, connections, average degree, clustering coefficients, and number of modules compared to the agricultural soils (Figure 2b). In contrast, for keystone fungi taxa, agricultural soil had the lowest number of nodes, connections, and average degrees, whereas wastewater treatment plant soil exhibited the highest values for these metrics, including clustering coefficients and modules (Figure 2d). Additionally, most nodes within the co-occurrence network patterns were positively correlated (Figure 2a,c), suggesting that keystone taxa tend to coexist in mutually beneficial relationships. Overall, the network structures at the hospital and sewage treatment plant sites were more complex than those of the agricultural and zoo soils (Figure 2 and Supplementary Figure S2).

### 3.3. Community Composition and Niche Width of Potential Keystone Taxa

**Figure 3:**
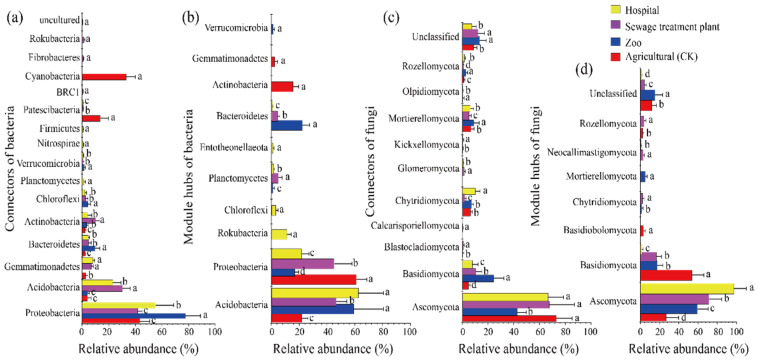
Differences in composition and abundance of soil bacterial (**a**,**b**) and fungal (**c**,**d**) keystone taxa at the phylum level in urban and peri-urban areas. Different capital letters indicate a significant difference (*p* < 0.05).

The keystone taxa exhibited variability across different taxonomic levels in urban and peri-urban areas. Overall species diversity was richer in urban ecosystems compared to pre-urban ecosystems (Supplementary Figure S3). Bacterial connectors and module hubs across all samples were categorized into 15 and 10 phyla, respectively. Proteobacteria emerged as the most abundant group (accounting for 54.37% of connectors and 35.89% of module hubs), followed by Acidobacteria (15.35% of connectors and 47.28% of module hubs) (Figure 3a,b).

For fungi, connectors and module hubs were classified into 10 and 7 distinct phyla (Supplementary Figure S3), respectively. *Ascomycota* was the most dominant group (62.33% of connectors and 63.79% of module hubs), followed by *Basidiomycota* (11.73% and 22.41%, respectively) (Figure 3c,d). These phyla—*Proteobacteria*, *Acidobacteria*, *Ascomycota*, and *Basidiomycota*—constituted a significant portion of the keystone taxa and showed distinct distribution patterns between urban and peri-urban areas (Figure 3). In this study, *Proteobacteria* was the most abundant bacterial key taxon across all four habitats, while Ascomycota was the most abundant fungal key taxon, both exceeding 40%.

**Figure 4:**
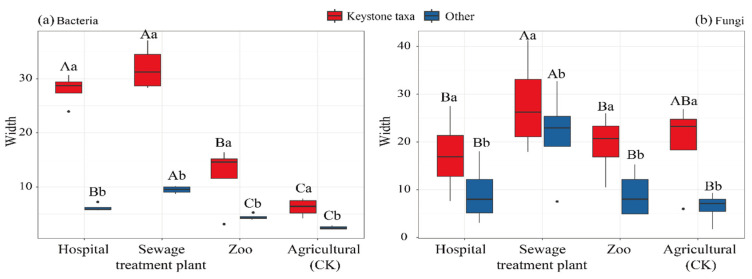
Niche widths of keystone microbial communities in urban and peri-urban soils. Different capital letters indicate a significant difference (*p* < 0.05) between keystone taxa urban and peri-urban soils. Different lower-case letters indicate a significant difference (*p* < 0.05) between keystone taxa from urban and peri-urban soils. H: hospital; S: sewage treatment plant; Z: zoo; A: agricultural.

Niche-width analysis demonstrated that both keystone bacterial and fungal taxa in urban and peri-urban soils possessed significantly broader niche width than other taxa (Figure 4). The niche width of keystone bacterial taxa was higher in urban environments, including hospitals, wastewater treatment plants, and zoos, compared to peri-urban, agricultural soils (Figure 4a). In contrast, the niche widths of keystone fungal taxa in urban and peri-urban soils did not show a consistent pattern (Figure 4b). For example, soil from the sewage treatment plants’ niche exhibited a significantly wider fungal niche than those from the hospital, zoo, and agricultural sites, while no significant differences were observed among the hospital, zoo, and agricultural soils.

### 3.4. Community Diversity and Stability of Keystone Taxa

**Figure 5:**
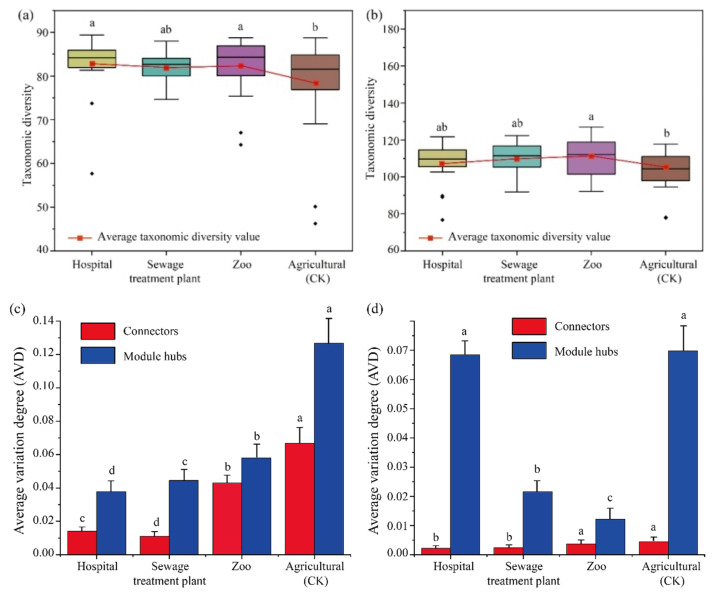
Diversity and average variation degree of the keystone bacterial and fungal communities. The taxonomic diversity of keystone bacteria (**a**) and fungi (**b**) in urban and peri-urban soils. The average variation degree (AVD) of keystone bacterial (**c**) and fungal (**d**) communities. Different capital letters indicate a significant difference (*p* < 0.05).

Taxonomic diversity analysis revealed that both bacterial and fungal diversity were greater in hospital, sewage treatment plant, and zoo soils compared to agricultural soils (Figure 5a,b). The average variation degree (AVD), an important indicator for evaluating microbiota stability [25], indicated significant differences in bacterial keystone between urban (hospitals, sewage treatment plants, and zoos) and peri-urban (agricultural) areas. The AVD of agricultural soils (CK) was significantly higher than that of urban areas (*p* < 0.05) (Figure 5c). Similarly, the AVD of fungal communities was highest in agricultural soils, indicating reduced stability of the soil microbial community in peri-urban soils compared to their urban counterparts (Figure 5d).

### 3.5. Functional Prediction of Keystone Taxa

**Figure 6:**
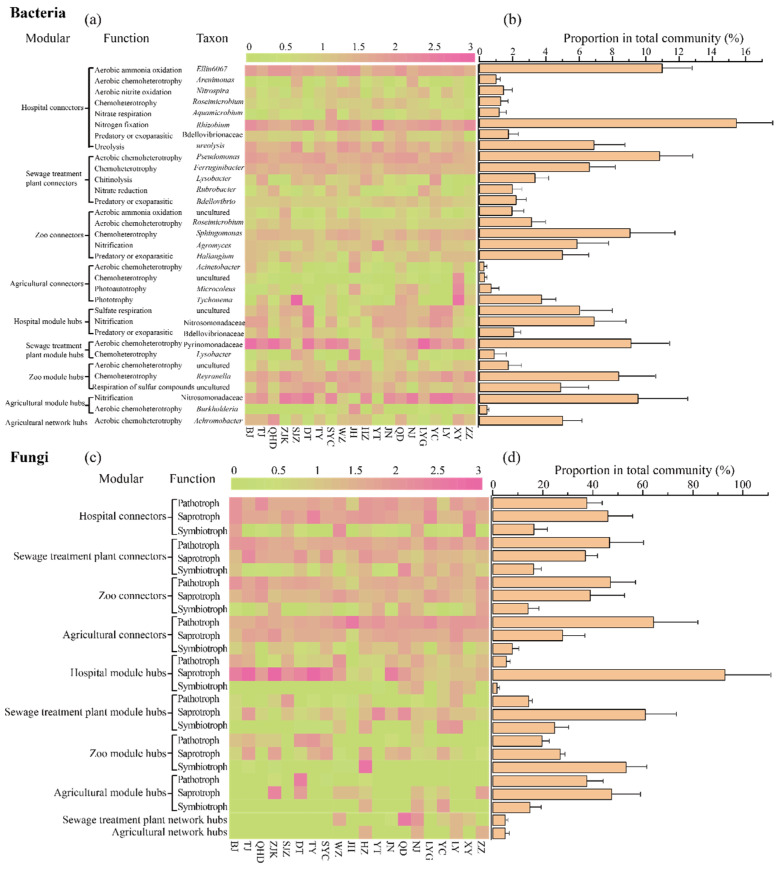
Taxonomic annotations of the keystone functions of bacteria and fungi. (**a**,**c**) are based on the relative abundances of different sample data from urban and peri-urban soils. The colors from green to purple indicate the relative abundance of each genus from low to high. Relative abundances of (**b**,**d**) correspond with genera based on genomic data. The error bar represents the standard deviation (n = 80).

Utilizing FAPROTAX and FUNGuild, we annotated the potential functions of keystone bacterial and fungal taxa, respectively (Figure 6). Keystone bacterial taxa were primarily associated with Chemoheterotrophy, nitration, and carbon and sulfur metabolism, with most taxa belonging to Proteobacteria. Among these, *Nitrospira* was the most common genus involved in soil nitrogen metabolism, followed by *Nitrosomonadaceae*, *Rhizobium*, *Rhizobacter*, *Aquamicrobium*, *Rubrobacter*, and *Agomyces* (Figure 6a). *Pseudomonas* was identified as the most common genus involved in soil carbon metabolism, followed by *Arenimonas*, *Roseimicrobium*, *Ferruginibacter*, *Sphingomonas*, *Acinetobacter*, an unclassified genus of Pyrinomonadaceae, *Lysobacter*, *Reyranella*, *Burkholderia*, and *Achromobacter* (Figure 6a). An unclassified genus from Desulfarculaceae was associated with sulfur cycling (Figure 6a).

Keystone fungal taxa predominantly performed saprotrophic, symbiotrophic, and pathotrophic functions. Notably, there were significant differences in the dominant functional groups between urban and peri-urban soils (Figure 6b,d). Specifically, bacterial communities in agricultural soils were dominated by nitrogen fixation and chemoheterotrophy, while fungal communities were dominated by pathogenic and saprophytic functions. In hospital soils, bacterial functions were dominated by ammonia oxidation and nitrogen fixation, while fungal communities were mainly pathotrophic. Wastewater treatment plant soils were characterized by bacterial chemoheterotrophy and fungal pathotrophic and saprotrophic functions. In zoo soils, bacterial communities primarily exhibited chemoheterotrophic functions, while fungal communities displayed both pathotrophic and symbiotrophic functions.

### 3.6. Linkages Between Microbial Community Stability and Keystone Taxa and Environmental Factors

**Figure 7:**
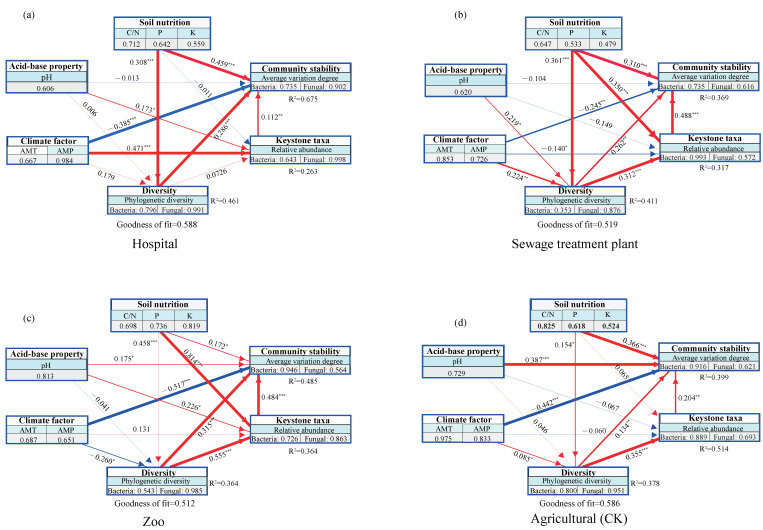
Contributions of biotic and abiotic factors to the stability of microbial communities. (**a**–**d**): Partial least squares path models (n = 80). Each oblong box represents a latent variable, and the parameters in each oblong box represent a manifest variable. Path coefficients and coefficients of determination (*R*^2^) are calculated after 1000 bootstraps. *, **, and *** represent *p* < 0.05, *p* < 0.01, and *p* < 0.001, respectively. Wider arrows indicate larger path coefficients, and red and blue lines indicate positive and negative correlations, respectively. The Goodness of Fit (GoF) values of PLS-PM in hospital, sewage treatment plant, zoo, and agricultural soils were 0.588, 0.519, 0.512, and 0.586, respectively.

Keystone taxa play an essential role in maintaining community stability and functional diversity. Urbanization influences keystone taxa and the diversity of bacterial and fungal communities, both directly or indirectly (*p* < 0.05 or *p* < 0.001). The Goodness of Fit (GoF) values of PLS-PM at the hospitals, sewage treatment plants, zoos, and agricultural sites were 0.588, 0.519, 0.512, and 0.586, respectively (Figure 7). A GoF > 0.5 is often considered acceptable. Values between 0.512 and 0.588 suggest a moderate to strong model fit. The magnitude of environmental impacts on microbial community diversity, keystone taxa, and overall stability varied between urban and peri-urban soils (Figure 7). Key taxa had a significant and positive influence on bacterial and fungal community stability; this positive effect was stronger in soils from the zoos (0.48) and sewage treatment plant (0.49) areas than in hospital and agricultural soils. Soil nutrients (i.e., C/N ratio, phosphorus, and potassium) exerted a stronger direct effect on the stability of keystone taxa in hospital soil compared to other sites. Climatic factors (i.e., mean annual precipitation [MAP] and mean annual temperature [MAT]) significantly and negatively influenced keystone taxa stability overall but had a significant positive effect on keystone taxa in hospital soils. The influence of pH on community stability was particularly pronounced in agricultural soils (*p* < 0.001), more so than in hospital, sewage treatment plant, and zoo soils (Figure 7d).

## 4. Discussion

Co-occurrence network analysis used to predict keystone taxa of soil fungi and bacteria offers valuable insights into community stability and potential responses to environmental disturbance [35]. In this study, keystone taxa were more diverse in hospital, sewage treatment plant, and zoo soils than in the control (agricultural) soils. This indicates that these urban environments support a richer diversity of microbial species compared to agricultural land. Proteobacteria and Acidobacteria (bacteria) along with Ascomycota and Basidiomycota (fungi) were the dominant keystone taxa in urban and peri-urban soils, and their abundance shifted with urbanization. This suggests that urbanization may drive convergence in keystone taxa composition between urban and peri-urban areas. However, at larger spatial scales, variation in keystone taxa abundance may increase structural differences in microbial communities across different sites [38].

In this study, network hubs were identified only in the co-occurrence networks of bacterial and fungal communities in peri-urban agricultural soils and sewage treatment plants and were absent in hospital and zoo soils. Additionally, agricultural soils had the lowest number of connected nodes, whereas sewage treatment plants and zoos exhibited the highest number of bacterial and fungal module hubs, respectively. These findings suggest that urbanization imposes ecological stress on soil microbes, promoting modular aggregations within microbial communities. This modularity enhances microbial community stability and leads to increased diversity of keystone microbes in urban greenspaces. There are a number of obstacles to inferring ecological relationships from sequencing data, such as difficulties in reliably measuring species abundance from sequence counts, technical biases, and difficulties in data interpretation brought on by intrinsic sample complexity [39]. The use of environmental sequencing, sometimes referred to as metagenomics, to comprehend organismal communities in diverse settings is growing, but deciphering the enormous volume of fragmented DNA from unidentified species is challenging [39]. However, previous research has shown that keystone species in microbial symbiotic networks may develop specific habitat preferences following anthropogenic disturbances, which can lead to the homogenization of microbial communities and a consequent decline in biodiversity [35,40,41,42]. By changing social networks, ecological processes, and particular community components like plant and microbial communities, the extent of urbanization has a substantial impact on community structure and function that extends beyond the effects on keystone species. Urbanization is a complex phenomenon that has the potential to alter many environmental conditions, upend established social institutions, and hasten species phenotypic changes [43]. Keystone species have an impact on many other organisms in an environment and are essential to preserving the stability and structure of an ecological community. Without them, the environment would either drastically change or vanish completely, making their presence crucial to the ecosystem. Their substantial impact on ecosystem structure, function, and biodiversity—which is abnormally large in relation to their own biomass or abundance—is the cause of this “overrepresentation”. The functions and effects of keystone species in an ecosystem are greatly influenced by abiotic variables. Compared to fungal keystone taxa, bacterial keystone taxa in subtropical forests are more impacted by abiotic variables, including soil and topography. The entire microbial functions and stability within the soils are greatly influenced by this increased correlation between keystone taxa and abiotic environments. Research indicates that keystone species mediate the indirect effects of abiotic environments and have direct effects on the formation of microbial communities, a function not seen in uncommon taxa [44]. In this study, agricultural soils under long-term crop rotation experiences had more severe anthropogenic disturbances than the other three types of urban soils, leading to a reduction in key microbial groups and decreased microbial diversity.

Network analysis was employed to identify highly correlated keystone microbial taxa in urban and peri-urban soils and to examine their interactions within microbial communities. Symbiotic species that are closely related within a community can cluster into modules, which significantly impacts ecosystem stability and function [45,46]. In this study, the microbial networks of soils from hospitals, sewage treatment plants, and zoos were found to be more complex than those of agricultural land. This complexity contributes to greater resistance to environmental disturbances and enhances community stability, although such communities may also be more sensitive to the influence of highly connected keystone taxa [47]. These findings underscore the importance of interspecific interactions among keystone taxa in promoting community stability. Urbanization has altered the structure of keystone microbial communities, reshaping the symbiotic organization of urban and peri-urban soil microbial networks. In conclusion, the study found that urbanization has increased soil bacterial and fungal diversity in urban areas relative to peri-urban areas. Additionally, AVD analysis showed that soil from hospitals, sewage treatment plants, and zoos exhibited high taxonomic diversity, whereas agricultural soils displayed lower diversity. These observations challenge the common view that urbanization generally reduces microbial diversity. The results suggest that microbial networks with a higher number of potential keystone taxa tend to be more stable. The diversity and stability of soil microbial communities vary in response to environmental changes, such as soil compaction, phosphorus enrichment, pH, land use, and vegetation cover [15,33]. Furthermore, keystone bacterial and fungal taxa in urban and peri-urban soils exhibited broader niche widths compared to other taxa. This is likely due to the continual environmental changes brought about by human activities in urban areas, which favor the survival of species with broader ecological range tolerances [48,49]. These keystone species can utilize a wide range of food resources and tolerate varied temperature conditions, often showing phenotypic plasticity behaviorally, physiologically, or morphologically.

Keystone taxa contribute to nutrient cycling by positively influencing the function of multiple microbial groups [50,51]. In this study, keystone bacterial taxa associated with chemoheterotrophy, nitrification, and carbon and sulfur metabolism showed significant variation in relative abundance between urban and peri-urban soils. Most keystone bacteria taxa belonged to the Proteobacteria phylum, consistent with findings from previous studies [52,53]. Notably, taxa such as *Nitrospira*, *Nitrosomonadaceae*, *Rhizobium*, *Rhizobacter*, and *Agomyces*, recognized for their roles in nitrogen cycling, were identified as potentially keystone taxa. Additionally, *Pseudomonas*, *Arenimonas*, *Roseimicrobium*, and *Ferruginibacter* were associated with carbon metabolism, while Desulfarculaceae (unclassified genus) were linked to sulfur cycle (Figure 6). For example, *Nitrospira* is an ammonia-oxidizing and nitrifying bacterium [54], and *Rhizobacter* and *Burkholderia* can function as nitrogen-fixing organisms [55,56]. The keystone fungal taxa were primarily from the phyla Ascomycota and Basidiomycota and functioned mainly as pathotrophs, symbiotrophs, and saprotrophs. In China’s peri-urban areas, agricultural soils are often used to cultivate vegetables, melons, and fruits with short harvest cycles, creating a high demand for nitrogen. This may be the primary factor contributing to the dominance of nitrogen-turnover and saprophytic functional groups in agricultural soil of peri-urban areas [54]. In contrast, microbial functional groups in the soils of hospitals, sewage treatment plants, and zoos were mainly fungal saprophytic and bacterial chemoheterotrophic. This suggests that although these environments are not nutrient-limited, they require numerous saprophytic taxa to degrade the substantial organic matter produced by green plants [55,56]. Environmental changes can alter species interactions, forcing species to adapt, migrate, be replaced, or face extinction [57,58].

The study also found that the factors influencing microbial community diversity vary when the communities are categorized by keystone taxa. The results indicate that microbial community stability is shaped by a combination of environmental and microbial factors, both directly and indirectly. Variables such as (MAP), carbon (C), nitrogen (N), phosphorus (P), potassium (K), microbial diversity, and keystone taxa are crucial in determining microbial community composition and stability (Figure 7). Keystone taxa in particular play a central role in determining the diversity and stability of urban soil microbial communities. Previous studies have shown that the distribution of soil bacterial communities is largely governed by moisture and C and N content [59,60]. In urban greenspaces, standardized management practices ensure that green plants do not lack water or C and N nutrients. The study found that soil nutrient levels, specifically carbon, nitrogen, phosphorus, and potassium, were higher in agricultural land compared to green soils from hospitals, sewage treatment plants, and zoos. These nutrients were also identified as key factors influencing the presence and dynamics of keystone microbial taxa. Anthropogenic activities, such as tillage soil compaction, phosphate fertilizer application, and pH regulation, significantly affect the assembly process of soil keystone microbial communities in agricultural environments [33,61]. In conclusion, urbanization alters the structural diversity and stability of keystone microbial communities [62,63].

## 5. Conclusions

Urbanization tends to enhance the diversity and stability of soil microbial communities. The co-occurrence networks of soil microorganisms in urban areas are more complex than those in peri-urban areas and are predominantly characterized by positive interactions. This suggests that interspecies cooperation contributes to greater community stability. Furthermore, keystone bacterial taxa are mainly involved in carbon, nitrogen, and sulfur metabolism, while keystone fungal taxa primarily perform saprophytic, symbiotic, and pathogenic functions. These functional keystone taxa provide a theoretical foundation for understanding changes in microbial communities and offer new insights into species interactions and the preservation of microbial community stability in urban ecosystems. These understandings can help manage urban soil sustainably, enhance ecosystem services, and direct microbiome-based strategies for pollution reduction, green infrastructure, and soil restoration. In order to better understand the roles of keystone taxa in the sustainability of urban ecosystems across various climates and geographical areas, future research should concentrate on how urban activities impact microbial networks and functions and make use of cutting-edge technologies like metagenomics.

## Data Availability

The original contributions presented in this study are included in the article/Supplementary Material. Further inquiries can be directed to the corresponding authors.

## Supplementary Materials

The following supporting information can be downloaded at https://www.mdpi.com/article/10.3390/microorganisms13081726/s1: Supplemental Figure S1: Geographical distribution of soil sampling sites in urban ecosystems. Triangles, rectangles, pentagrams and circles represent agricultural, hospital, sewage treatment plant and zoo sampling sites, respectively; Supplemental Figure S2: Bacterial (a), fungal (c) co-occurrence network and their topological properties (b and d) in urban and peri-urban areas; Supplemental Figure S3: Composition of soil keystone bacterial (a,b) and fungal communities (c,d) at the classification level in urban and peri-urban areas; Supplemental Table S1: Geographical distribution and basic information of sampling sites in urban and peri-urban areas; Supplemental Table S2: Characteristics of environmental variables in urban and peri-urban areas.
