# Supplementary material for: Deciphering Soil Keystone Microbial Taxa: Structural Diversity and Co-Occurrence Patterns from Peri-Urban to Urban Landscapes
Source: Microorganisms. 2025 Jul 24;13(8):1726. doi: 10.3390/microorganisms13081726 (PMC12388032; doi:10.3390/microorganisms13081726)

Supplemental Figure S1. Geographical distribution of soil sampling sites in urban ecosystems. Triangles, rectangles, pentagrams and circles represent agricultural, hospital, sewage treatment plant and zoo sampling sites, respectively.

Supplemental Figure S2. Bacterial (a), fungal (c) co-occurrence network and their topological properties (b and d) in urban and peri-urban areas.

Supplemental Figure S3. Composition of soil keystone bacterial (a, b) and fungal communities (c, d) at the classification level in urban and peri-urban areas.

Supplemental Figure S1

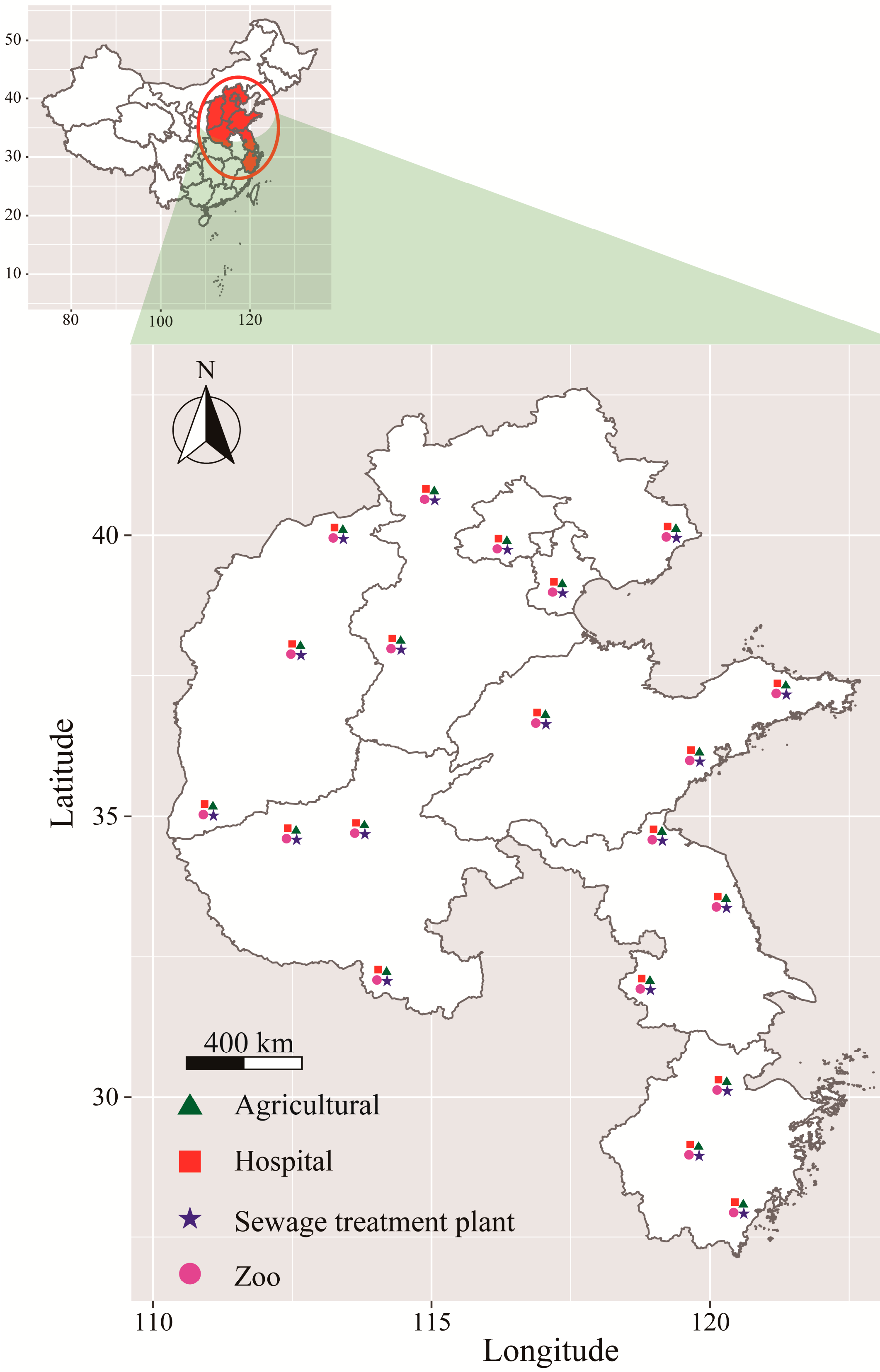

Supplemental Figure S2

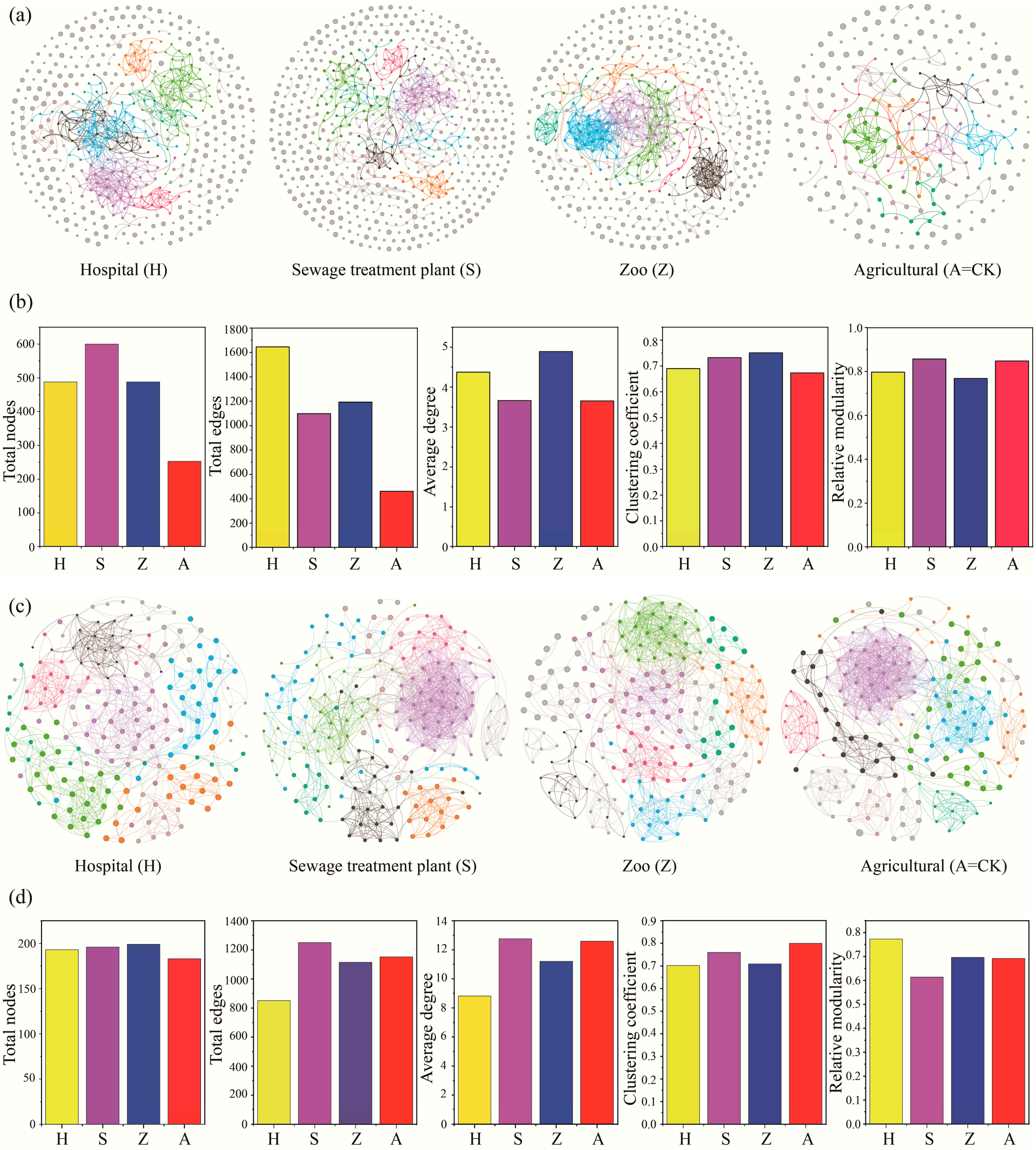

Supplemental Figure S3

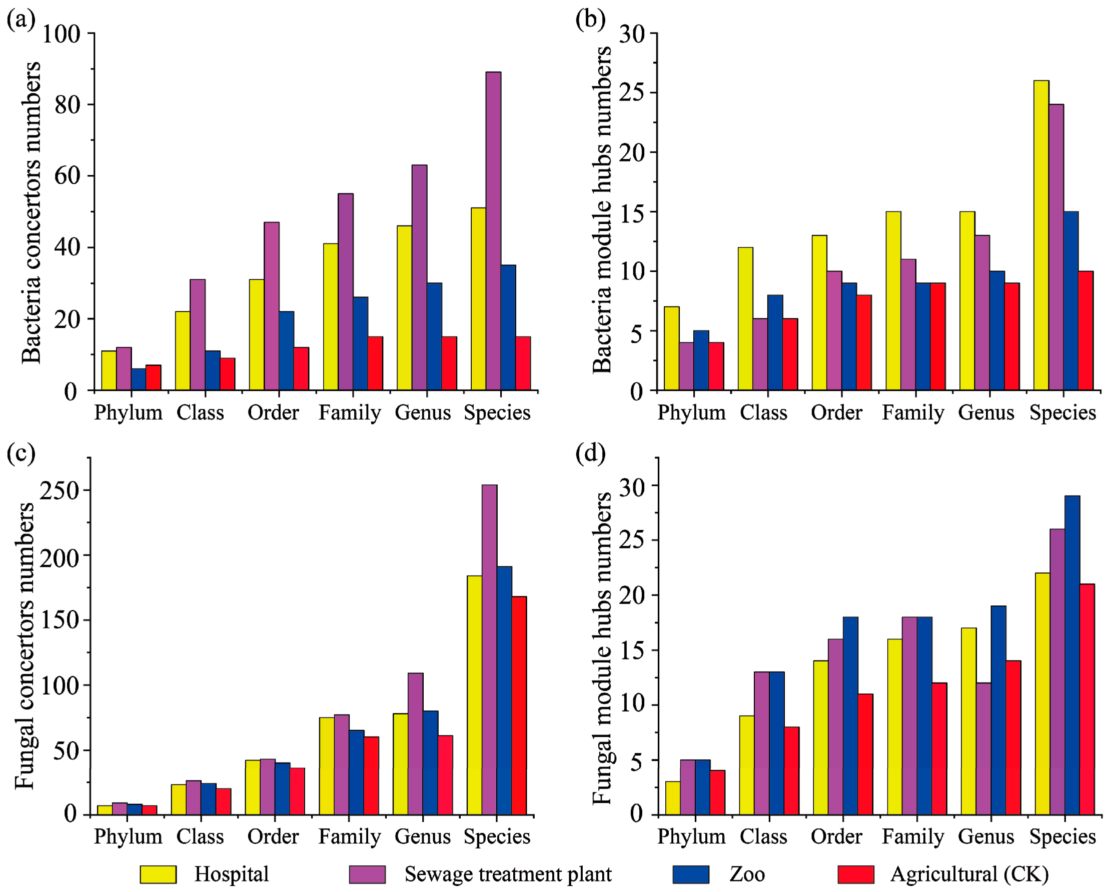

Supplement: Supplementary file 1 [file microorganisms-13-01726-s001.zip › microorganisms-3697348 Supplemental Figure.pdf]
