# Supplementary material for: Deciphering Soil Keystone Microbial Taxa: Structural Diversity and Co-Occurrence Patterns from Peri-Urban to Urban Landscapes
Source: Microorganisms. 2025 Jul 24;13(8):1726. doi: 10.3390/microorganisms13081726 (PMC12388032; doi:10.3390/microorganisms13081726)
Supplement: Supplementary file 1 [file microorganisms-13-01726-s001.zip › microorganisms-3697348 Supplemental Table.pdf]

Supplemental Table S1 Geographical distribution and basic information of sampling sites in urban and peri-urban areas.

Province	Sample code	Latitude (ºN)	Longitude (ºE)	Altitude (m)
Beijing (BJ)	BJA	39º75'54"	116º19'34"	35
	BJH	39º93'53"	116º34'91"	35
	BJS	39º75'56"	116º19'00"	34
	BJZ	39º93'92"	116º33'60"	36
Tianjin (TJ)	TJA	38º97'41"	117º39'53"	42
	TJH	39º14'60"	117º14'11"	37
	TJS	38º96'65"	117º40'03"	41
	TJZ	39º08'32"	117º15'80"	36
Hebei (HB)	QHDA	39º94'81"	119º66'64"	21
	QHDH	39º93'66"	119º59'62"	18
	QHDS	39º94'81"	119º66'64"	21
	QHDZ	39º85'90"	119º51'46"	24
	ZJKA	40º71'33"	114º88'78"	688
	ZJKH	40º82'54"	114º86'54"	796
	ZJKS	40º71'13"	114º88'90"	689
	ZJKZ	40º60'02"	115º04'84"	626
	SJZA	38º04'43"	114º37'46"	109
	SJZH	38º05'77"	114º45'81"	102
	SJZS	38º04'52"	114º37'84"	111
	SJZZ	38º06'60"	114º31'06"	143
Shanxi (SX)	DTA	40º93'77"	113º52'38"	1078
	DTH	40º07'37"	113º33'94"	1097
	DTS	40º04'51"	113º34'69"	1081
	DTZ	40º09'76"	113º27'87"	1105
	TYA	37º93'77"	112º52'38"	888
	TYH	37º86'90"	112º57'08"	899
	TYS	37º93'37"	112º51'75"	906
	TYZ	37º90'88"	112º56'91"	922
	SYCA	35º10'37"	110º98'23"	500
	SYCH	35º03'48"	111º02'79"	906
	SYCS	35º05'80"	111º08'55"	450
	SYCZ	35º10'74"	111º07'35"	471
Zhejiang (ZJ)	WZA	27º99'50"	120º59'22"	43
	WZH	27º97'23"	120º62'12"	53
	WZS	27º99'43"	120º59'32"	41
	WZZ	28º00'10"	120º63'42"	112
	JHA	28º98'02"	119º63'71"	110
	JHH	29º10'93"	119º65'44"	115
	JHS	29º07'89"	119º60'21"	214

	JHZ	28º97'83"	119º63'72"	120
	HZA	30º15'34"	120º29'06"	58
	HZH	30º20'54"	120º19'56"	45
	HZS	30º18'15"	120º16'72"	53
	HZZ	30º21'57"	120º12'94"	66
	YTA	37º58'92"	121º35'61"	97
	YTH	37º53'56"	121º38'60"	83
	YTS	37º58'92"	121º35'61"	69
	YTZ	37º51'94"	121º38'26"	153
	JNA	36º73'79"	116º99'85"	110
Shandong	JNH	36º70'08"	116º98'60"	115
(SD)	JNS	36º72'05"	116º94'71"	214
	JNZ	36º70'41"	116º98'20"	120
	QDA	36º05'13"	120º14'51"	79
	QDH	35.98'52"	120º15'10"	76
	QDS	36º11'61"	120º09'91"	57
	QDZ	35º98'74"	120º06'49"	71
	NJA	32º17'04"	118º82'53"	66
	NJH	32º09'67"	118º79'43"	85
	NJS	31º95'93"	118º73'55"	79
	NJZ	32º09'93"	118º81'56"	91
	LYGA	34º70'55"	119º10'48"	94
Jiangshu	LYGH	34º61'25"	119º18'86"	106
(JS)	LYGS	34º70'54"	119º10'49"	96
	LYGZ	34º59'90"	119º16'76"	103
	YCA	33º35'11"	120º24'38"	82
	YCH	33º39'39"	120º11'50"	85
	YCS	33º35'11"	120º24'38"	83
	YCZ	32º12'98"	118º60'42"	87
	LYA	34º63'72"	112º53'39"	217
	LYH	34º67'03"	112º42'32"	249
	LYS	34º63'16"	112º53'42"	219
	LYZ	34º66'82"	112º41'53"	242
	XYA	32º14'88"	114º14'42"	137
Henan	XYH	32º13'47"	114º07'75"	137
(HN)	XYS	32º10'59"	114º15'15"	135
	XYZ	32º12'05"	114º06'37"	125
	ZZA	34º77'55"	113º83'03"	193
	ZZH	34º77'70"	113º65'90"	181
	ZZS	34º78'36"	113º61'41"	208
	ZZZ	34º78'91"	113º67'80"	181

BJ: Beijing, BJH: Beijing hospital, BJS: Beijing sewage treatment plant, BJZ: Beijing zoo, BJA: Beijing agricultural; TJ: Tianjin, TJH: Tianjin hospital, TJS: Tianjin sewage treatment plant, TJZ: Tianjin zoo, TJA: Tianjin agricultural; HB: Hebei, QHD: Qinhuangdao, QHDH: Qinhuangdao hospital, QHDS: Qinhuangdao sewage treatment plant, QHDZ: Qinhuangdao zoo, QHDA: Qinhuangdao agricultural; ZJK: Zhangjiakou, ZJKH:

Zhangjiakou hospital, ZJKS: Zhangjiakou sewage treatment plant, ZJKZ: Zhangjiakou zoo, ZJKA: Zhangjiakou agricultural; SJZ: Shijiazhuang, SJZH: Shijiazhuang hospital, SJZS: Shijiazhuang sewage treatment plant, SJZZ: Shijiazhuang zoo, SJZA: Shijiazhuang agricultural; SX: Shanxi, DT: Datong, DTH: Datong hospital, DTS: Datong sewage treatment plant, DTZ: Datong zoo, DTA: Datong agricultural; TY: Taiyuan, TYH: Taiyuan hospital, TYS: Taiyuan sewage treatment plant, TYZ: Taiyuan zoo, TYA: Taiyuan agricultural; SYC: Yuncheng, SYCH: Yuncheng hospital, SYCS: Yuncheng sewage treatment plant, SYCZ: Yuncheng zoo, SYA: Yuncheng agricultural; ZJ: Zhejiang, WZ: Wenzhou, WZH: Wenzhou hospital, WZS: Wenzhou sewage treatment plant, WZZ: Wenzhou zoo, WZA:Wenzhou agricultural; JH: Jinhua, JHH: Jinhua hospital, JHS: Jinhua sewage treatment plant, JHZ: Jinhua zoo, JHA: Jinhua agricultural; HZ: Hangzhou, HZH: Hangzhou hospital, HZS: Hangzhou sewage treatment plant, HZZ: Hangzhou zoo, HZA: Hangzhou agricultural; SD: Shandong, SDH: Shandong hospital, SDS: Shandong sewage treatment plant, SDZ: Shandong zoo, SDA: Shandong agricultural; YT: Yantai, YTH: Yantai hospital, YTS: Yantai sewage treatment plant, YTZ: Yantai zoo, YTA: Yantai agricultural; JN: Jinan, JNH: Jinan hospital, JNS: Jinan sewage treatment plant, JNZ: Jinan zoo, JNA: Jinan agricultural; QD: Qingdao, QDH: Qingdao hospital, QDS: Qingdao sewage treatment plant, QDZ: Qingdao zoo, QDA: Qingdao agricultural; JS: Jiangshu, JSH: Jiangshu hospital, JSS: Jiangshu sewage treatment plant, JSZ: Jiangshu zoo, JSA: Jiangshu agricultural; NJ: Nanjing, NJH: Nanjing hospital, NJS: Nanjing sewage treatment plant, NJZ: Nanjing zoo, NJA: Nanjing agricultural; LYG: Lianyungang, LYGH: Lianyungang hospital, LYGS: Lianyungang sewage treatment plant, LYGZ: Lianyungang zoo, LYGA: Lianyungang agricultural; YC: Yancheng, YCH: Yancheng hospital, YCS: Yancheng sewage treatment plant, YCZ: Yancheng zoo, YCA: Yancheng agricultural; HN: Henan, HNH: Henan hospital, HNS: Henan sewage treatment plant, HNZ: Henan zoo, HNA: Henan agricultural; LY: Luoyang, LYH: Luoyang hospital, LYS: Luoyang sewage treatment plant, LYZ: Luoyang zoo, LYA: Luoyang agricultural; XY: Xinyang, XYH: Xinyang hospital, XYS: Xinyang sewage treatment plant, XYZ: Xinyang zoo, XYA: Xinyang agricultural; ZZ: Zhengzhou. ZZH: Zhengzhou hospital, ZZS: Zhengzhou sewage treatment plant, ZZZ: Zhengzhou zoo, ZZA: Zhengzhou agricultural.

Supplemental Table S2 Characteristics of environmental variables in urban and peri-urban areas.

Province	Site	MAT (℃)	MAP (mm)	pH	TN (g/kg)	TP (g/kg)	TK (g/kg)	SOC (g/kg)	AN (mg/kg)	AP (mg/kg)	AK (mg/kg)
	BJA	13.49±3.72	67.23±20.18	6.70±0.11	0.91±0.045	0.046±0.0017	21.77±5.63	106.76±3.56	134.19±2.06	265.43±12.34	190.04±8.03
Beijing	BJH	13.18±3.45	64.00±19.68	6.35±0.09	0.75±0.032	0.027±0.0013	9.91±1.38	67.82±2.14	22.91±1.95	215.11±6.24	72.51±4.54
(BJ)	BJS	13.49±3.16	66.26±21.73	6.67±0.02	0.41±0.017	0.017±0.0006	9.91±2.10	64.96±1.32	44.47±2.73	26.58±2.13	174.37±5.69
	BJZ	13.16±2.99	60.91±22.01	6.13±0.21	0.47±0.0025	0.035±0.0022	2.83±0.86	6.81±0.96	26.28±3.85	137.24±8.24	91.67±0.95
	TJA	13.19±2.25	64.90±7.93	6.35±0.64	1.12±0.0067	0.134±0.0002	24.55±3.59	149.61±9.09	293.09±19.65	148.00±7.16	253.59±12.54
Tianjin	TJH	13.08±2.16	72.50±8.19	6.33±0.52	0.47±0.0012	0.025±0.0002	20.71±2.19	79.71±8.31	24.93±2.07	17.56±2.10	109.08±1.25
(TJ)	TJS	13.19±1.86	62.87±7.22	6.30±0.51	0.66±0.0036	0.030±0.0001	10.92±1.63	88.60±7.05	229.76±13.02	14.16±3.84	146.51±12.06
	TJZ	12.77±1.64	72.40±5.37	6.63±0.66	0.75±0.0048	0.037±0.0003	9.30±1.87	74.85±6.32	78.16±2.07	47.60±0.64	147.38±8.62
	QHDA	11.62±1.99	76.05±16.68	6.63±0.64	1.03±0.059	0.044±0.0002	21.93±5.28	110.52±4.32	227.73±19.32	424.72±15.18	187.43±13.06
	QHDH	11.62±1.85	73.27±16.53	6.43±0.58	0.42±0.027	0.021±0.0001	11.82±3.73	78.20±9.74	47.16±3.49	151.28±2.03	135.19±1.85
	QHDS	11.62±1.47	73.27±15.49	6.60±0.43	0.57±0.037	0.032±0.0004	16.77±4.18	100.28±15.36	55.25±4.79	318.23±17.16	91.67±1.02
	QHDZ	8.44±1.08	90.04±10.21	6.52±0.71	0.39±0.004	0.014±0.0001	18.29±2.99	84.60±2.63	28.30±6.23	76.37±3.09	65.55±9.64
	ZJKA	9.35±2.49	36.59±8.39	6.47±0.27	1.16±0.08	0.148±0.0012	30.61±10.03	166.12±22.07	264.79±7.15	513.83±22.14	318.01±3.47
Hebei	ZJKH	7.78±1.17	33.26±7.04	6.72±0.08	0.62±0.02	0.036±0.001	23.74±3.04	51.35±1.68	33.69±5.16	33.85±2.78	118.65±3.06
(HB)	ZJKS	9.35±2.09	35.33±8.13	6.28±0.17	0.97±0.007	0.062±0.008	17.08±4.17	68.70±3.09	93.65±3.07	173.42±19.05	265.78±9.85
	ZJKZ	5.84±0.83	39.38±10.07	6.20±0.04	0.98±0.003	0.039±0.004	24.96±2.37	73.20±1.28	272.20±15.49	336.79±24.18	127.36±1.65
	SJZA	15.10±2.08	67.39±18.47	6.87±0.02	0.81±0.0019	0.071±0.0003	25.86±2.64	136.40±9.31	214.37±12.65	194.69±11.16	150.86±6.45
	SJZH	15.26±2.62	64.97±17.19	8.53±0.09	0.44±0.0073	0.023±0.0001	9.80±1.07	8.87±1.03	37.73±7.42	19.43±2.97	136.93±5.49
	SJZS	15.10±1.74	66.56±18.32	6.57±0.04	0.72±0.0008	0.049±0.0005	9.60±1.16	48.37±2.52	49.86±3.28	29.56±1.26	137.81±16.24
	SJZZ	15.07±2.02	63.70±20.01	6.82±0.09	0.52±0.007	0.029±0.0001	3.95±0.63	26.36±1.94	59.97±8.66	34.16±1.75	79.48±9.42
	DTA	8.56±2.45	34.12±7.10	6.65±0.07	0.86±0.004	0.042±0.0005	16.52±3.19	112.96±6.24	144.86±14.62	83.40±3.04	154.35±6.42
	DTH	8.60±2.38	34.13±6.29	6.82±0.10	0.58±0.02	0.022±0.0001	9.91±1.05	14.00±2.65	73.44±1.48	8.21±0.85	111.69±8.51
	DTS	8.60±2.33	34.13±7.37	6.73±0.06	0.42±0.01	0.020±0.0001	8.89±1.07	48.10±5.67	58.62±4.76	10.22±2.09	69.90±8.46
	DTZ	6.16±1.67	30.04±6.85	6.63±0.01	0.49±0.007	0.031±0.0002	4.45±0.94	76.40±1.49	98.37±1.07	53.26±1.06	148.25±12.37
Shanxi	TYA	11.89±1.09	39.31±7.29	6.48±0.03	0.92±0.002	0.068±0.0001	24.96±1.19	99.01±3.41	191.63±3.07	282.73±21.64	197.87±14.03
(SX)	TYH	12.17±2.81	38.98±7.19	6.48±0.02	0.45±0.0013	0.021±0.0002	15.36±2.48	71.03±4.36	45.82±1.98	9.42±1.74	12.44±3.02
	TYS	11.89±2.31	38.49±8.06	6.33±0.04	0.89±0.0024	0.034±0.0003	9.40±2.64	71.05±1.02	88.26±1.67	33.12±2.13	128.23±2.57
	TYZ	10.33±3.07	38.71±7.77	6.38±0.01	0.51±0.001	0.043±0.0001	10.01±1.33	60.96±1.11	88.94±5.46	213.87±15.34	156.09±6.04
	SYCA	15.26±1.93	70.71±6.29	6.88±0.05	1.06±0.07	0.087±0.0002	21.82±1.71	145.63±12.51	227.73±9.24	340.90±12.19	217.03±11.06
	SYCH	15.51±2.08	73.56±10.13	6.02±0.04	0.46±0.003	0.018±0.0001	19.20±1.19	62.66±14.02	38.40±2.03	42.24±3.29	109.08±10.08

	SYCS	15.51±1.57	73.56±9.62	6.65±0.03	0.71±0.004	0.056±0.0006	9.50±2.20	17.36±3.95	72.77±2.82	7.94±1.03	71.64±3.52
	SYCZ	13.43±2.36	73.02±8.26	6.35±0.02	0.46±0.001	0.057±0.0001	12.33±1.04	74.59±3.06	76.81±1.35	231.43±19.53	56.84±6.27
	WZA	19.16±3.38	217.02±39.73	6.87±0.05	1.16±0.036	0.063±0.0001	25.56±5.03	152.72±16.17	159.68±11.08	362.90±22.06	180.46±9.23
	WZH	19.31±3.09	210.25±40.16	6.38±0.04	0.75±0.0019	0.026±0.0001	9.50±2.64	38.98±3.93	78.83±9.46	114.50±9.05	146.51±11.57
	WZS	19.16±4.19	217.02±38.27	5.65±0.017	0.76±0.0062	0.049±0.0004	9.20±3.07	115.71±11.26	171.81±21.38	44.19±2.41	88.18±8.00
	WZZ	16.53±1.08	222.92±41.08	4.87±0.015	0.52±0.0027	0.020±0.0001	21.22±1.17	11.21±2.02	108.48±1.35	267.70±12.84	163.92±11.94
	JHA	17.95±2.06	185.75±28.51	6.48±0.06	0.80±0.0031	0.070±0.0006	18.29±0.31	93.29±5.04	95.00±3.09	371.22±15.19	121.26±2.07
Zhejiang (ZJ)	JHH	16.49±3.05	179.76±27.16	3.27±0.63	0.67±0.0017	0.033±0.0001	11.32±2.006	35.27±1.29	148.23±1.03	144.63±11.01	76.87±4.34
	JHS	19.09±4.08	166.59±25.09	6.55±0.34	0.67±0.0033	0.062±0.0005	17.48±0.08	63.79±2.06	135.43±4.52	415.87±23.09	106.47±8.08
	JHZ	17.56±1.19	207.85±22.17	4.82±0.57	0.55±0.0014	0.016±0.0001	13.84±3.34	74.62±1.16	125.32±2.36	215.43±15.46	94.28±6.27
	HZA	18.23±2.27	157.34±20.11	6.23±0.03	0.95±0.0026	0.082±0.0004	18.79±2.19	134.71±17.16	117.24±2.86	312.08±17.05	96.02±3.46
	HZH	18.20±2.95	149.21±13.48	6.35±0.21	0.78±0.0021	0.025±0.0001	9.10±2.06	94.43±2.03	97.02±1.91	124.27±9.64	52.49±1.75
	HZS	18.25±3.64	155.18±15.73	6.22±0.33	0.96±0.0001	0.025±0.0001	16.17±1.30	86.76±4.35	104.43±2.64	42.05±2.27	63.81±1.93
	HZZ	17.93±3.07	149.41±12.06	6.03±0.02	0.49±0.008	0.026±0.0001	16.27±2.04	116.43±4.61	30.99±2.26	258.20±11.38	79.48±2.58
	YTA	13.35±1.07	58.89±9.32	6.47±0.08	0.84±0.0062	0.064±0.0007	18.39±1.15	88.20±3.16	174.79±8.06	411.64±13.19	118.65±10.37
	YTH	13.35±3.12	58.53±10.48	6.37±0.07	0.37±0.0054	0.018±0.0001	10.31±3.07	42.54±2.92	272.88±3.50	208.93±6.57	64.68±4.65
	YTS	13.35±2.03	58.53±11.37	7.12±0.01	0.36±0.0001	0.020±0.0001	12.73±3.52	56.08±1.07	14.82±4.31	39.05±5.64	69.03±1.35
	YTZ	13.23±3.43	60.94±13.57	6.60±0.06	0.74±0.004	0.027±0.0001	18.79±1.76	66.07±1.09	60.64±1.08	114.13±6.24	42.91±5.64
	JNA	14.77±2.07	75.55±12.09	6.55±0.21	0.97±0.005	0.081±0.0054	21.99±2.05	159.08±6.29	259.40±4.95	317.31±7.25	183.07±12.04
Shandong (SD)	JNH	14.76±3.02	67.52±10.96	6.42±0.57	0.60±0.0011	0.018±0.0001	9.60±1.43	87.39±3.49	113.87±8.34	38.97±3.46	176.11±3.69
	JNS	14.76±2.96	67.52±11.22	6.25±0.09	0.80±0.0032	0.040±0.0003	16.57±3.55	173.48±4.30	70.07±9.06	30.72±1.28	115.17±11.04
	JNZ	14.75±3.08	66.33±13.65	6.47±0.11	0.61±0.001	0.026±0.0001	8.79±2.03	60.99±1.64	194.72±3.10	267.23±13.57	135.19±3.65
	QDA	14.11±2.64	73.22±8.14	6.73±0.24	0.96±0.0064	0.094±0.0047	28.69±2.06	113.60±16.07	155.47±9.84	403.01±25.23	157.83±8.58
	QDH	14.06±2.55	74.61±12.84	5.17±0.14	0.57±0.0047	0.062±0.0021	10.21±0.12	36.35±4.43	97.02±11.30	355.37±11.49	89.92±1.74
	QDS	14.14±2.71	72.12±11.73	6.80±0.06	0.52±0.0001	0.015±0.0001	23.95±7.05	88.52±11.02	109.82±16.83	206.37±12.32	44.66±2.63
	QDZ	14.14±1.06	72.12±12.06	5.92±0.07	0.53±0.0036	0.039±0.008	11.72±2.64	44.88±5.67	24.26±1.22	299.85±11.04	131.71±3.87
	NJA	17.06±3.20	111.93±12.37	6.37±0.18	0.85±0.0018	0.143±0.024	24.05±5.02	103.58±8.34	195.39±9.35	232.42±15.43	98.63±2.57
	NJH	17.11±2.07	110.89±8.96	6.65±0.02	0.67±0.0003	0.025±0.0011	11.82±4.13	26.87±2.28	60.64±2.13	134.97±9.06	69.03±1.87
	NJS	17.15±2.11	113.43±12.37	6.58±0.03	0.84±0.0049	0.121±0.095	7.88±0.32	95.80±9.94	115.89±4.08	92.61±2.42	78.61±1.23
	NJZ	16.72±1.63	108.05±13.39	6.28±0.09	0.54±0.0062	0.017±0.0001	16.87±1.06	65.45±1.20	76.81±1.83	154.29±10.23	88.18±2.01
	LYGA	15.17±1.07	99.54±15.37	6.34±0.38	1.16±0.051	0.052±0.0002	20.21±2.01	104.53±6.15	233.80±4.69	252.86±9.05	237.05±4.58
Jiangshu (JS)	LYGH	15.26±3.08	90.68±20.11	6.17±0.05	0.93±0.0014	0.020±0.0001	9.80±2.74	3.95±0.94	95.00±6.09	192.98±10.10	96.02±2.06
	LYGS	15.17±2.21	99.24±15.09	6.60±0.03	1.09±0.037	0.028±0.0002	7.58±0.99	72.94±1.34	114.54±2.50	153.64±1.32	109.08±6.52
	LYGZ	15.06±2.81	98.52±17.39	6.13±0.01	0.93±0.006	0.028±0.0002	11.32±1.04	7.93±1.05	70.75±3.27	143.53±6.57	48.14±1.34
	YCA	16.10±1.96	108.09±18.07	6.53±0.03	0.88±0.092	0.067±0.0025	21.72±2.54	109.30±5.19	120.67±1.43	411.64±15.37	175.24±4.89
	YCH	16.04±2.37	111.15±18.08	6.32±0.05	0.56±0.008	0.031±0.0001	4.25±0.48	68.83±1.32	117.91±3.09	294.35±12.83	76.87±2.25
	YCS	16.10±3.02	107.68±10.08	6.65±0.06	0.51±0.0064	0.029±0.0001	9.00±1.03	76.30±5.73	35.04±2.45	11.93±2.03	105.59±2.03
	YCZ	16.96±3.18	106.75±9.37	6.68±0.02	0.82±0.0043	0.019±0.0001	16.67±1.11	49.33±1.06	90.96±8.43	74.05±3.61	102.98±5.23
	LYA	16.63±2.84	80.25±10.58	6.05±0.27	1.02±0.0031	0.139±0.076	25.86±1.07	139.58±14.18	175.01±13.62	463.52±5.17	266.65±2.34
	LYH	16.58±1.08	80.36±9.62	6.08±0.06	0.64±0.001	0.031±0.0015	12.73±1.30	102.58±5.06	43.80±1.32	89.58±5.67	111.69±1.67
	LYS	16.63±2.09	79.89±12.35	6.02±0.01	0.71±0.008	0.026±0.0001	15.56±3.48	68.32±1.07	165.75±6.24	39.88±3.62	191.78±5.84
	LYZ	15.77±1.33	82.21±8.37	5.93±0.02	0.52±0.002	0.036±0.0002	8.69±1.16	87.98±1.25	151.60±18.76	315.08±5.16	137.81±4.06
	XYA	16.68±2.07	116.57±19.64	6.83±0.35	0.90±0.0017	0.063±0.0037	20.33±3.07	101.43±5.61	288.37±12.39	416.36±16.25	143.90±2.65
Henan (HN)	XYH	16.62±1.19	108.83±11.03	6.68±0.02	0.48±0.0031	0.037±0.0022	11.93±2.56	73.34±2.04	86.24±1.05	15.50±1.96	63.81±2.25
	XYS	16.68±1.20	115.47±15.07	6.27±0.01	0.88±0.0019	0.027±0.00019	21.02±6.07	106.81±1.92	301.18±9.14	212.19±6.52	127.36±6.35
	XYZ	16.67±1.38	118.97±18.11	6.28±0.06	0.55±0.0012	0.029±0.00011	10.92±3.41	51.18±0.02	73.44±2.06	302.65±7.18	128.23±13.26
	ZZA	16.26±3.08	86.67±8.29	6.28±0.07	0.91±0.03	0.082±0.00061	22.83±0.74	145.11±3.85	192.02±9.16	63.72±1.40	184.82±10.34
	ZZH	16.24±1.07	88.57±10.34	6.22±0.02	0.46±0.0034	0.026±0.0001	20.41±1.03	176.12±6.03	30.32±2.15	28.79±0.80	47.27±3.12
	ZZS	16.24±3.09	88.57±9.16	6.30±0.08	0.79±0.0011	0.032±0.0001	9.91±0.44	81.58±3.92	43.80±0.67	98.05±7.47	119.52±6.95
	ZZZ	17.93±3.66	149.41±16.82	6.35±0.01	0.89±0.006	0.005±0.00001	16.98±2.15	89.02±0.48	11.45±0.02	91.65±5.32	98.63±3.65

BJ: Beijing, BJH: Beijing hospital, BJS: Beijing sewage treatment plant, BJZ: Beijing zoo, BJA: Beijing agricultural; TJ: Tianjin, TJH: Tianjin hospital, TJS: Tianjin sewage treatment plant, TJZ: Tianjin zoo, TJA: Tianjin agricultural; HB: Hebei, QHD: Qinhuangdao, QHDH: Qinhuangdao hospital, QHDS: Qinhuangdao sewage treatment plant, QHDZ: Qinhuangdao zoo, QHDA: Qinhuangdao agricultural; ZJK: Zhangjiakou, ZJKH: Zhangjiakou hospital, ZJKS: Zhangjiakou sewage treatment plant, ZJKZ: Zhangjiakou zoo, ZJKA: Zhangjiakou agricultural; SJZ: Shijiazhuang, SJZH: Shijiazhuang hospital, SJZS: Shijiazhuang sewage treatment plant, SJZZ: Shijiazhuang zoo, SJZA: Shijiazhuang agricultural; SX: Shanxi, DT: Datong, DTH: Datong hospital, DTS: Datong sewage treatment plant, DTZ: Datong zoo, DTA: Datong agricultural; TY: Taiyuan, TYH: Taiyuan hospital, TYS: Taiyuan sewage treatment plant, TYZ: Taiyuan zoo, TYA: Taiyuan agricultural; SYC: Yuncheng, SYCH: Yuncheng

hospital, SYCS: Yuncheng sewage treatment plant, SYCZ: Yuncheng zoo, SYA: Yuncheng agricultural; ZJ: Zhejiang, WZ: Wenzhou, WZH: Wenzhou hospital, WZS: Wenzhou sewage treatment plant, WZZ: Wenzhou zoo, WZA:Wenzhou agricultural; JH: Jinhua, JHH: Jinhua hospital, JHS: Jinhua sewage treatment plant, JHZ: Jinhua zoo, JHA: Jinhua agricultural; HZ: Hangzhou, HZH: Hangzhou hospital, HZS: Hangzhou sewage treatment plant, HZZ: Hangzhou zoo, HZA: Hangzhou agricultural; SD: Shandong, SDH: Shandong hospital, SDS: Shandong sewage treatment plant, SDZ: Shandong zoo, SDA: Shandong agricultural; YT: Yantai, YTH: Yantai hospital, YTS: Yantai sewage treatment plant, YTZ: Yantai zoo, YTA: Yantai agricultural; JN: Jinan, JNH: Jinan hospital, JNS: Jinan sewage treatment plant, JNZ: Jinan zoo, JNA: Jinan agricultural; QD: Qingdao, QDH: Qingdao hospital, QDS: Qingdao sewage treatment plant, QDZ: Qingdao zoo, QDA: Qingdao agricultural; JS: Jiangshu, JSH: Jiangshu hospital, JSS: Jiangshu sewage treatment plant, JSZ: Jiangshu zoo, JSA: Jiangshu agricultural; NJ: Nanjing, NJH: Nanjing hospital, NJS: Nanjing sewage treatment plant, NJZ: Nanjing zoo, NJA: Nanjing agricultural; LYG: Lianyungang, LYGH: Lianyungang hospital, LYGS: Lianyungang sewage treatment plant, LYGZ: Lianyungang zoo, LYGA: Lianyungang agricultural; YC: Yancheng, YCH: Yancheng hospital, YCS: Yancheng sewage treatment plant, YCZ: Yancheng zoo, YCA: Yancheng agricultural; HN: Henan, HNH: Henan hospital, HNS: Henan sewage treatment plant, HNZ: Henan zoo, HNA: Henan agricultural; LY: Luoyang, LYH: Luoyang hospital, LYS: Luoyang sewage treatment plant, LYZ: Luoyang zoo, LYA: Luoyang agricultural; XY: Xinyang, XYH: Xinyang hospital, XYS: Xinyang sewage treatment plant, XYZ: Xinyang zoo, XYA: Xinyang agricultural; ZZ: Zhengzhou. ZZH: Zhengzhou hospital, ZZS: Zhengzhou sewage treatment plant, ZZZ: Zhengzhou zoo, ZZA: Zhengzhou agricultural, MAP: mean annual precipitation, MAT: mean annual temperature, pH: Soil acidity and alkalinity, TN: Total nitrogen, TP: Total phosphorus, TK: Total potassium, SOC: Organic carbon, AN: Available nitrogen, AP: Available phosphorus, AK: Available potassium.
